# *Aeromonas caviae*-Associated Severe Bloody Diarrhea

**DOI:** 10.1155/2023/4966879

**Published:** 2023-10-09

**Authors:** Xiaolan Tang, Anuoluwa Oyetoran, Tyler Jones, Christopher Bray

**Affiliations:** ^1^University of Central Florida College of Medicine, Graduate Medical Education, 6850 Lake Nona Blvd, Orlando, FL 32827, USA; ^2^HCA Florida North Florida Hospital, Internal Medicine Residency Program, 6500 W Newberry Rd, Gainesville, FL 32605, USA

## Abstract

*Aeromonas* species are capable of inducing severe infections in both immunocompetent and immunocompromised individuals. Gastroenteritis is the most common infection associated with *Aeromonas* species in humans. We report a rare case of *Aeromonas caviae* severe gastroenteritis and bloody diarrhea that led to the development of sepsis in a 45-year-old female with no history of immunocompromising conditions. This patient required extensive medical support which included blood transfusions and antibiotics. Fortunately, with appropriate diagnostic measures and targeted antibiotic therapy, her symptoms resolved. *Aeromonas* species are becoming increasingly frequent among the pathogens isolated from patients suffering from gastroenteritis. As such, it is becoming increasingly important for clinicians to consider this pathogen when working up their patients for diarrhea.

## 1. Introduction

There are a few culprits considered to be the primary causes of gastroenteritis. There are however instances during which an unusual pathogen makes its way into the gastrointestinal (GI) tract and wreaks havoc. Here, we discuss such a case. *Aeromonas caviae* is the offending bacterium in this case. This is a species growing in importance with respect to GI tract infections. As such, we aim to illustrate its presentation in this case, the means by which we isolated the organism and the significance of its ever-growing pertinence in our approach toward treating GI infections. To accomplish this objective, we will investigate the case of one young woman who suffered such an infection. While incidents of this infection have been reported, they typically involved an immunocompromised patient. Our patient was not immunocompromised, and yet she developed sepsis from this infection. Following will be an account of the means by which a shift in the microbiome of our communities is altering our approach to treating infections in patients of a similar presentation.

## 2. Case Presentation

A 45-year-old female with a past medical history of bipolar disorder, chronic tobacco use, and a Roux-en-Y gastric bypass presented to our emergency department following three weeks of nonbloody diarrhea. She did not have any immunocompromising conditions. It had become bloody a day prior to presentation. This was associated with abdominal pain and cramping, nausea, and subjective fevers. Symptoms had started in the early winter. She denied recent travel, nonsteroidal anti-inflammatory drug use, or use of any antiplatelets or anticoagulants. Prior to presentation, the patient reported to her primary care physician's office and stool cultures were performed. These were positive for norovirus.

Surgical history was significant for cholecystectomy, gastric bypass surgery, a hysterectomy, and a liver biopsy. The patient denied any family history of inflammatory bowel disease or colorectal cancer. Home medications included trazodone and venlafaxine.

On physical examination, her temperature was 36.6 degrees Celsius, heart rate was 113 beats per minute, respiratory rate was 18 breaths per minute, and her blood pressure was 114/67 millimeters of mercury. No rash was appreciated on our examination. The abdomen was soft and tender to palpation in the hypogastric and epigastric regions. A complete blood count was significant for leukocytosis of 29,200 cells per cubic millimeter (cmm), a hemoglobin of 9.3 grams per deciliter (g/dl), and thrombocytosis of 833 per cmm. A comprehensive metabolic panel revealed a blood urea nitrogen of 23 milligrams per deciliter (mg/dl) and a creatinine of 0.51 mg/dl. There were no abnormalities on her liver function panel.

**Figure 1:**
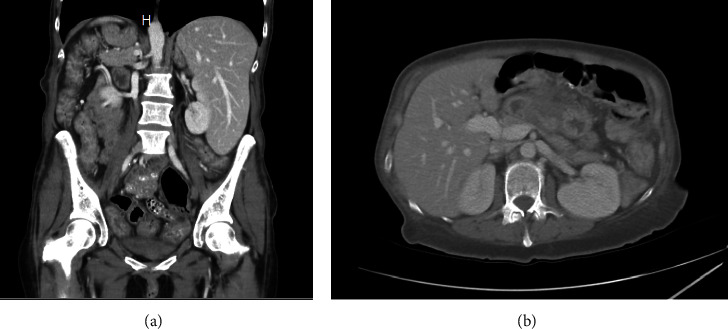
(a, b) CT scan showing mild colitis in the transverse and descending colon.

Computed tomography of the abdomen and pelvis revealed stable postsurgical changes with mild inflammatory changes suggestive of mild colitis, as shown in figures 1(a) and 1(b).

The patient met sepsis criteria with tachycardia and leukocytosis secondary to colitis. She was fluid resuscitated and started on empiric piperacillin and tazobactam in the emergency department.

Her bloody diarrhea resolved on her second day of admission. Antibiotics were then switched to azithromycin for a narrower spectrum of coverage. On day three of admission, the patient had four episodes of bloody diarrhea with her hemoglobin decreasing to 6.6 g/dl. She remained hemodynamically stable and was transfused two units of packed red blood cells, with a posttransfusion hemoglobin of 9.8 g/dl. Stool cultures acquired on admission grew *Aeromonas caviae* on day four of hospitalization. This culture was sensitive to ciprofloxacin, which she received for the following seven days. We monitored her for another two days. By the sixth day of admission, her symptoms had resolved and her hemoglobin had remained stable. She was discharged home with the remaining course of her antibiotics, as well as instructions to follow-up closely with her primary care physician.

## 3. Discussion

A number of important identifying factors are worth paying attention to with respect to *Aeromonas* species (spp.). For starters, they are both catalase and oxidase positive. These are also Gram-negative rods that are facultatively anaerobic. Brackish and fresh water are typical environments in which to find these organisms. They have unfortunately been found in drinking water as well [1, 2]. They can grow in colder environments (zero to 42 degrees Celsius) but are being isolated in warmer months as well. Caviae, hydrophila, and sobria are the most frequently seen organisms when it comes to *Aeromonas* infections [3, 4].

Travelers' diarrhea is becoming more frequent among those visiting parts of Asia, South America, and Africa. In fact, gastroenteritis is the most common infection caused by *Aeromonas* spp. in humans. While watery diarrhea is the most common symptom with which patients present, far more severe symptoms are by no means uncommon. The watery stool can progress to being a chronic issue, even mucousy and bloody in some. *Aeromonas* can in fact infect various organ symptoms. This is especially true in those with comorbid conditions. For example, spontaneous bacterial peritonitis can be seen in those with liver cirrhosis. Intra-abdominal infections and abscesses may occur as well. Even extraordinarily fatal conditions such as osteomyelitis and meningitis can be seen in those with immunocompromising conditions [5–7]. There are no specific clinical characteristics of an *Aeromonas* infection. However, a thorough history exemplifying the above characteristic certainly points towards this infection.

Infections most often occur through ingestion of water from unfiltered sources, such as rivers and lakes. With that been said, there are also cases of this organism being identified in chlorinated hospital tap water [8]. There are other additional vectors for infection. Seafood, undercooked meat, and dairy products have been known to act as sources for infection [9–12]. What is worse is that there are mechanisms that allow *Aeromonas* spp. to become resistant to various antibiotics. For example, they create enterotoxins and cytotoxins [13]. In addition, infections are becoming increasingly correlated with malignancies [14, 15].

There is something clinicians can do to immediately address this growing threat. As of now, *Aeromonas* spp. are not typically tested for in microbiology labs. Because of the growing incidence of *Aeromonas* gastroenteritis [16], it is incumbent upon clinicians to specifically request testing for *Aeromonas* in stool cultures, when they suspect it as a possible etiology. While *Aeromonas* infections are generally speaking self-limiting illnesses, it is important to follow patients and their clinical course. A failure to improve may be an indication for further supportive and even antibiotic therapy. This is more likely to be the case in those with severe diarrhea, especially in immunocompromised patients [17]. As of today, there is no specific risk factor we are able to identify that is specific to this patient developing this illness. Although she had undergone gastric sleeve surgery, our literature review did not produce articles citing this as a risk factor for gastroenteritis. There is a correlation with small intestinal bacteria overgrow (SIBO) and intra-abdominal surgeries. SIBO was not however diagnosed in our patient. During our literature review, we did not find articles citing gastroenteritis as being more common in those with a history of intra-abdominal surgeries.

## 4. Conclusion

A 45-year-old female with no history of immunocompromising conditions presented for bloody diarrhea. Through thorough history and laboratory evaluation, she was diagnosed with *Aeromonas* gastroenteritis. This had led to the development of sepsis. Her infection resolved with appropriate antibiotics. The most salient point of this case is the diagnostic means that were undertaken. Appropriate history evaluation and attention to detail were essential to what led to the targeted cultures necessary to identify the pathogen. *Aeromonas* spp. are becoming increasingly frequent among the pathogens isolated from patients suffering from gastroenteritis. Including this bacterium within the differential for potential causes for gastroenteritis is becoming more important with each passing year. This portends a change in the microbiome that medical doctors will need to address. With shifting prevalence in pathogens comes a shift in the need for research. Whether this will require an alteration in the way labs test for antibiotic sensitivities, or the degree to which we culture stool, urine, blood, and so on to further elucidate the specific pathogen within our populations is yet to be determined.

## Data Availability

No underlying data were collected or produced in this study.
